# Potential for Drug-Drug Interactions between Antiretrovirals and HCV Direct Acting Antivirals in a Large Cohort of HIV/HCV Coinfected Patients

**DOI:** 10.1371/journal.pone.0141164

**Published:** 2015-10-21

**Authors:** Isabelle Poizot-Martin, Alissa Naqvi, Véronique Obry-Roguet, Marc-Antoine Valantin, Lise Cuzin, Eric Billaud, Antoine Cheret, David Rey, Christine Jacomet, Claudine Duvivier, Pascal Pugliese, Pierre Pradat, Laurent Cotte, Hepadat'AIDS Study Group

**Affiliations:** 1 Aix-Marseille University, APHM Hôpital Sainte-Marguerite, Immuno-Hematology Clinic, Marseille, France; 2 Inserm U912 (SESSTIM), Marseille, France; 3 CHU de Nice, Hôpital Archet 1, Service de Maladies Infectieuses, Unité de Virologie Clinique, Nice, France; 4 Sorbonne Universités, UPMC Université Paris 06, UMR_S 1136, Institut Pierre Louis d’Epidémiologie et de Santé Publique, F-75013, Paris, France; 5 APHP, Groupe hospitalier Pitié-Salpêtrière-Université Pierre et Marie Curie, Service de Maladies Infectieuses et Tropicales, Paris, France; 6 INSERM, UMR 1027, Toulouse, F-31000, France; Université de Toulouse III, Toulouse, F-31000, France; CHU Toulouse, COREVIH Toulouse, F-31000, France; 7 Service des maladies infectieuses, CHU, Hôtel-Dieu, Nantes, France; 8 Centre Hospitalier de Tourcoing, Service Universitaire de Maladies Infectieuses, Tourcoing, France; 9 CHU Bicêtre, Service de Médecine Interne, Le Kremlin-Bicêtre, France; 10 Laboratoire de Virologie, Hôpital Necker EA 7327 Université Paris Descartes, Paris, France; 11 Hôpitaux Universitaires de Strasbourg, Center for HIV care, Strasbourg, France; 12 CHU Clermont-Ferrand, Service des Maladies Infectieuses et tropicales, Clermont-Ferrand, France; 13 APHP-Hopital Necker, Service de Maladies Infectieuses et Tropicales, Centre d'Infectiologie Necker-Pasteur IHU Imagine, Paris, France; 14 Institut Pasteur, Centre Médical—Centre d'Infectiologie Necker-Pasteur, Paris, France; 15 Hospices civils de Lyon, Hôpital de la Croix Rousse, Service d’Hépatologie, Centre de recherche clinique, Lyon, France; 16 Inserm U1052, Lyon, France; 17 Hospices civils de Lyon, Hôpital de la Croix Rousse, Service des maladies infectieuses, Lyon, France; Kaohsiung Medical University Hospital, Kaohsiung Medical University, TAIWAN

## Abstract

**Objectives:**

Development of direct acting antivirals (DAA) offers new benefits for patients with chronic hepatitis C. The combination of these drugs with antiretroviral treatment (cART) is a real challenge in HIV/HCV coinfected patients. The aim of this study was to describe potential drug-drug interactions between DAAs and antiretroviral drugs in a cohort of HIV/HCV coinfected patients.

**Methods:**

Cross-sectional study of all HIV/HCV coinfected patients attending at least one visit in 2012 in the multicenter French Dat’AIDS cohort. A simulation of drug-drug interactions between antiretroviral treatment and DAAs available in 2015 was performed.

**Results:**

Of 16,634 HIV-infected patients, 2,511 had detectable anti-HCV antibodies, of whom 1,196 had a detectable HCV-RNA and were not receiving HCV treatment at the time of analysis. 97.1% of these patients were receiving cART and 81.2% had a plasma HIV RNA <50 copies/mL. cART included combinations of nucleoside reverse transcriptase inhibitors with a boosted protease inhibitor in 43.6%, a non-nucleoside reverse transcriptase inhibitor in 17.3%, an integrase inhibitor in 15.4% and various combinations or antiretroviral drugs in 23.7% of patients. A previous treatment against HCV had been administered in 64.4% of patients. Contraindicated associations/potential interactions were expected between cART and respectively sofosbuvir (0.2%/0%), sofosbuvir/ledipasvir (0.2%/67.6%), daclatasvir (0%/49.4%), ombitasvir/boosted paritaprevir (with or without dasabuvir) (34.4%/52.2%) and simeprevir (78.8%/0%).

**Conclusions:**

Significant potential drug-drug interactions are expected between cART and the currently available DAAs in the majority of HIV/HCV coinfected patients. Sofosbuvir/ledipasvir and sofosbuvir/daclatasvir with or without ribavirin appeared the most suitable combinations in our population. A close collaboration between hepatologists and HIV/AIDS specialists appears necessary for the management of HCV treatment concomitantly to cART.

## Introduction

In industrialized countries, hepatitis C virus (HCV) coinfection concerns about one-third of HIV-infected people [1] with an estimated prevalence in France of 16% to 18% [2]. Beside classical risk factors like age or alcoholism, HIV infection is known to favor liver disease progression. One-third of HIV-infected patients with chronic hepatitis C infection are indeed expected to progress to cirrhosis within less than 20 years, HIV/HCV coinfected individuals having a three-fold higher risk of progression to cirrhosis or decompensated liver disease than HCV monoinfected patients [3, 4].

Until recently, treatment of chronic HCV infection was restricted to pegylated interferon (PEG-IFN) and ribavirin, leading to poor response rates and bad tolerability [5]. After 2011, the association of first-generation HCV protease inhibitors (boceprevir or telaprevir) with PEG-IFN and ribavirin significantly increased the response rates in both naive and pre-treated patients leading to sustained virological response (SVR) rates similar to those observed in HCV mono-infected patients [6–8]. However, the tolerability of these regimens was poor, due to the cumulated toxicity of these first-generation direct acting antiviral agents (DAAs) and those of IFN and ribavirin.

The development of next-generation DAAs offers new perspectives with the availability of all-oral, better tolerated, IFN-free regimens with impressive virological results both in HCV monoinfected patients [9–13] and in HIV/HCV coinfected patients [14–16]. As a result, both American and European guidelines now recommend that HIV/HCV coinfected patients should be treated the same way as HCV monoinfected patients [17, 18]. However, these new combinations introduce new challenges in terms of interactions with combined antiretroviral treatment (cART) and/or treatment for comorbidities [19–21], leading both guidelines to emphasize the importance of identifying and managing these interactions.

So far, few data are available regarding the antiretroviral regimens currently prescribed in HIV/HCV co-infected patients. The present study was conducted to describe a large cohort of HIV/HCV coinfected patients enrolled in a French multicenter cohort of HIV-infected patients, and to emphasize the specificity of this population regarding potential interactions between DAAs and antiretroviral drugs.

## Material and Methods

### The Dat’AIDS cohort

A cross-sectional observational study was conducted at the end of 2012 using the multicenter Dat’AIDS cohort. The Dat’AIDS Cohort represents a collaboration between 10 major French HIV treatment centers using a common electronic medical record for the follow-up of HIV-, hepatitis B virus (HBV)- and HCV-infected adults (NADIS^®^ [Fedialis Medica, Marly le Roi, France]), corresponding to a representative sample of the French infected population regarding potential inter-region disparities [22]. Patient-related data are recorded during medical visits in a structured database, allowing the use of the database for clinical, epidemiological or therapeutic studies. Data quality is ensured by automated checks during data capture, regular controls, annual assessments, and *ad hoc* processes before any scientific analysis is performed.

### Study population

Data from all HIV/HCV coinfected patients attending at least one visit in the participating centers in 2012 were collected, including demographics, biological data related to HIV and HCV infections and current combination of antiretroviral treatment (cART).

### Data collected

Demographic data, last available CD4 cell count, last available HIV-RNA, HCV genotype (the most recent one in case of reinfection) and the last antiretroviral treatment were recorded.

Liver fibrosis was evaluated by liver biopsy and/or elastometry (Fibroscan^®^) and/or Fibrotest^®^, and results were converted into METAVIR fibrosis score equivalent. For patients with successive fibrosis evaluations, the last score was retained for the study. For patients evaluated by several methods at the same time, the fibrosis score determined by liver biopsy was kept as a priority against elastometry, the latter being prioritized against Fibrotest^®^. Fibroscan^®^-based assessment was considered valid if the Inter Quartile Range (IQR) was ≤ 30% and the success rate ≥50%. The fibrosis score was defined as a function of liver stiffness as: ≤7 kPa: F0-F1; 7–14.5 kPa: F2-F3; ≥14.5 kPa: F4.

### HCV treatment status

HCV treatment status was defined as naive, spontaneous cure, sustained virological response (SVR), virological failure and reinfection. A patient was considered as previously treated if he had received either standard or PEG-IFN, with or without ribavirin at least once before the last visit. For analysis of potential drug-drug interactions with available DAAs, the last antiretroviral treatment received was considered.

### Potential drug-drug interactions

Potential drug-drug interactions between available DAAs in 2015 and the different antiretroviral drugs received by each patient at the last follow-up visit were simulated according to the most recent literature data [18] and using the University of Liverpool DDI tool [19]. DAAs under consideration were boceprevir (BOC), daclatasvir (DCV), ledipasvir/sofosbuvir (LED/SOF), ombitasvir/ritonavir boosted paritaprevir (OBV/PTVr); dasabuvir (DSV); simeprevir (SMV); sofosbuvir (SOF); telaprevir (TVR). Potential interactions with PEG-IFN and ribavirin were also considered for comparison. Interactions were classified as 1) no clinically significant interaction expected; 2) potential interaction that may require close monitoring, alteration of drug dosage or timing of administration; 3) contraindicated association, these drugs should not be coadministered.

### Statistical analysis

Discrete variables are presented as number of cases and percentages, and continuous variables are presented as median values and IQR. Characteristics between naïve and pretreated patients were compared using Pearson’s Chi-square test for categorical parameters and a t-test for continuous parameters. For all analyses, a two-tailed significance testing and a significance level of 0.05 were used. Statistical analysis was performed using SPSS version 19 for Windows (SPSS Inc., Chicago, IL, USA).

### Ethical considerations

This cohort was approved by the French “Commission Nationale Informatique et Liberté” (Registration number: 2001/762876/nadiscnil.doc) and all patients signed an informed consent before being included in the database.

## Results

### Patients’ characteristics

**Fig 1:**
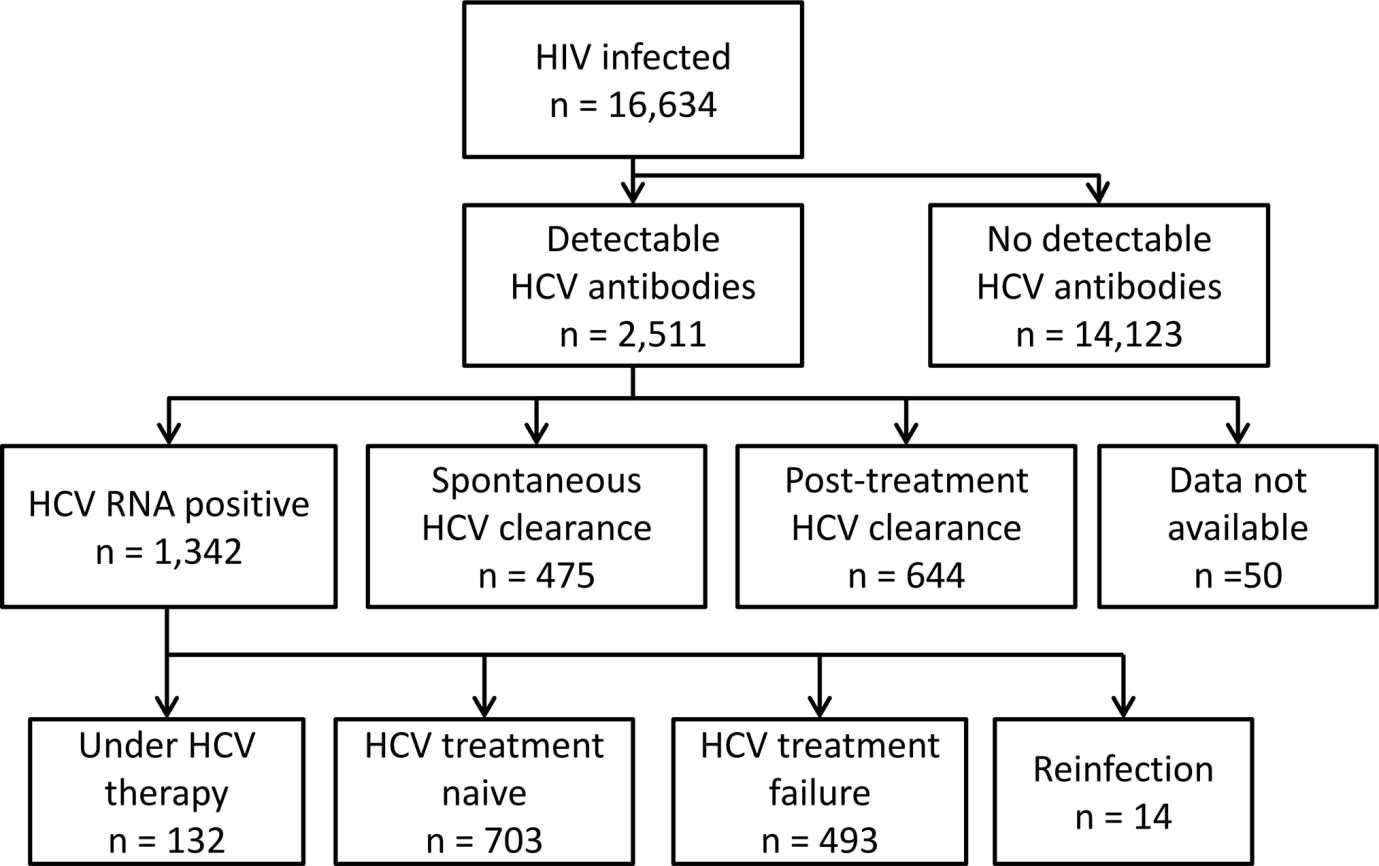
Flow chart of patient analysis.

Of 16,634 HIV-infected patients who attended at least one visit in the participating centers in 2012, 2,503 patients with HIV-1 infection and 8 patients with HIV-2 infection were HCV seropositive, corresponding to an overall prevalence of 15.1%. Among HIV/HCV coinfected patients, 475 patients (18.9%) cleared HCV spontaneously whereas 644 achieved viral clearance after treatment (25.6%), leading to an overall cure rate of 45.5% (Fig 1). Of the 1,972 patients who did not spontaneously cured HCV and who were not reinfected, 1,269 (64.4%) had already been previously treated or were under treatment at the time of analysis. Demographics and baseline characteristics of HIV monoinfected and HIV/HCV coinfected patients are summarized in S1 Table.

Of 2,511 HIV/HCV coinfected patients, 72% were males and median age was 49 years. Median CD4 cell count was 561 and 88% of patients had an HIV RNA level below 50 copies/mL. A total of 1342 patients (54%) had a detectable HCV-RNA at the time of analysis (naïve 52.4%, treatment failure 36.7%, under HCV treatment 9.8%, reinfection 1%). Characteristics of naive and pretreated patients are described in Table 1. Naïve patients had a shorter follow-up of their HIV infection, a shorter exposure to ART and had received less cART lines than pre-treated patients. HCV genotype 1 largely predominated in both groups, followed by genotype 4 and 3. Genotype 1 was found in 53.9% of naïve patients vs 64.3% of pre-treated patients (p = 0.0005). Severe fibrosis or cirrhosis (METAVIR F3-F4) was found in 435 of 968 patients with a fibrosis evaluation (44.9%). Fibrosis evaluation was significantly less severe in naïve patients than in pre-treated patients. Fibrosis scores according to the mode of fibrosis assessment are presented in S2 Table. Naïve patients were more prone to report a current or a past alcohol consumption, while no significant difference was observed regarding substance abuse between both groups.

**Table 1:** Characteristics of 1196 patients with a detectable HCV-RNA, according to HCV-treatment status (patients under HCV treatment at the time of analysis and patients with HCV reinfection excluded).

Characteristics	Naive patients (n = 703)	Treatment-experienced patients (n = 493)	p
Male, n *(%)*	495 *(70*.*4)*	358 *(72*.*6)*	0.436
Age (years), median [IQR]	49.0 [45.0–52.0]	50.0 [47.0–53.0]	<0.001
Alcohol consumption, n *(%)*	n = 546	n = 435	0.007
None	181 *(33*.*2)*	161 *(37*.*0)*	
Former	85 *(15*.*6)*	92 *(21*.*1)*	
Current	280 *(51*.*3)*	182 *(41*.*8)*	
Substance abuse, n *(%)*	n = 470	n = 369	0.07
None	136 *(28*.*9)*	112 *(30*.*4)*	
Former	169 *(36*.*0)*	153 *(41*.*5)*	
Opiates substitution	86 *(18*.*3)*	46 *(12*.*5)*	
Current	79 *(16*.*8)*	53 *(14*.*4)*	
Follow-up of HIV infection (years), median [IQR]	21 [13–25]	23 [17–26]	< 0.001
CDC stage C, n *(%)*	213 *(30*.*2)*	139 *(28*.*2)*	0.432
Nadir CD4 (cells/mm^3^), median [IQR]	172 [69–273]	157 [70–240]	0.164
Current CD4 (cells/mm^3^), median [IQR]	513 [305–741]	608 [394–813]	<0.001
HIV viral load <50 copies/ml, n *(%)*	542 *(77*.*1)*	429 *(87*.*0)*	<0.001
Under cART, n *(%)*	676 *(96*.*2)*	485 *(98*.*4)*	0.035
cART stopped, n *(%)*	9 *(1*.*3)*	3 *(0*.*6)*	<0.001
cART exposure (years), median [IQR]	13.8 [6.2–17.0]	15.9 [12.3–18.4]	<0.001
Number of cART line, median [IQR]	5 [3–9]	8 [5–12]	<0.001
cART regimen, n *(%)*			0.005
2NRTI +1 bPI	318 *(47*.*0)*	188 *(38*.*8)*	
2NRTI+ 1 NNRTI	123 *(18*.*2)*	78 *(16*.*1)*	
2NRTI+ 1 INSTI	93 *(13*.*8)*	86 *(17*.*7)*	
Other combinations	142 *(21*.*0)*	133 *(27*.*4)*	
HCV RNA	n = 667	n = 484	
HCV RNA (log UI/mL), median [IQR]	6.1 [5.5–6.6]	6.1 [5.5–6.5]	0.113
HCV genotype, n *(%)*	n = 644	n = 485	0.012
1 unspecified	14 *(2*.*2)*	11 *(2*.*3)*	
1a	267 *(41*.*5)*	236 *(48*.*7)*	
1b	66 *(10*.*2)*	65 *(13*.*4)*	
2	17 *(2*.*6)*	7 *(1*.*4)*	
3	119 *(18*.*5)*	57 *(11*.*7)*	
4	158 *(24*.*5)*	107 *(22*.*1)*	
5	1 *(0*.*1)*	1 *(0*.*2)*	
6	2 *(0*.*3)*	1 *(0*.*2)*	
Fibrosis Score, n *(%)*	n = 525	n = 443	<0.001
F0-F1	279 *(53*.*2)*	152 *(34*.*3)*	
F2	54 *(10*.*3)*	48 *(10*.*8)*	
F3	115 *(21*.*9)*	119 *(26*.*8)*	
F4	77 *(14*.*7)*	48 *(10*.*8)*	
Creatinine clearance*	n = 643	n = 461	
Median [IQR]	94.6 [76.8–114.8]	92.5 [76.2–113.5]	
Clearance <30mL/min, n *(%)*	7 *(1*.*1)*	7 *(1*.*5)*	

CDC: Centers for Disease Control; IQR: interquartile range; ART: antiretroviral treatment; NRTI: nucleoside/nucleotide reverse transcriptase inhibitor; bPI: ritonavir-boosted protease inhibitor; NNRTI: non-nucleoside reverse transcriptase inhibitor; INSTI: integrase strand transfer inhibitor.

* Creatinine clearance estimated using the Cockcroft-Gault Equation: creatinine clearance = (140 –age (years)) / weight (kg) * 1.23 (male) or * 1.04 (female)

### HIV therapy

Almost all patients (97.1%) were receiving cART at the time of the study, leading to an undetectable HIV viral load in 77.1% of HCV naïve and 87.0% of HCV pre-treated patients. Two hundred and twenty different antiretroviral combinations were prescribed. Combinations of 2 nucleoside reverse transcriptase inhibitors (NRTI) and 1 ritonavir-boosted protease inhibitor (bPI) were prescribed in 43.6% of the patients, combinations of 2 NRTIs and 1 non-nucleoside reverse transcriptase inhibitor (NNRTI) in 17.3% of the patients, combinations of 2 NRTIs and 1 integrase strand transfer inhibitor (INSTI) in 15.4% of the patients and other combinations of 3 or more classes of drugs or atypical combinations such as combinations of bPI and INSTI or NNRTI and INSTI in 23.7% of the patients. Naïve patients received more frequently bPI combinations and pre-treated patients were more prone to receive 3 classes or atypical combinations. Among the 132 patients receiving HCV treatment at that time, 96.2% were under cART. INSTI combinations appeared more frequently prescribed in these patients than in the 1196 untreated patients (26.8%) while 43.3% were receiving bPI combinations, 13.4% NNRTI combinations and 16.5% other combinations. Among these 132 patients, 27 (20.5%) were receiving first-generation HCV protease inhibitors (boceprevir 7; telaprevir 20) and 105 (79.5%) were treated with PEG-IFN/ribavirin without DAA. Atypical cART combinations were prescribed in only 3.7% of them, while the distribution of other combinations appeared quite similar (bPI combinations 55.6%; NNRTI combinations 11.1%; INSTI combinations 29.6%).

### Potential drug-drug interactions

**Fig 2:**
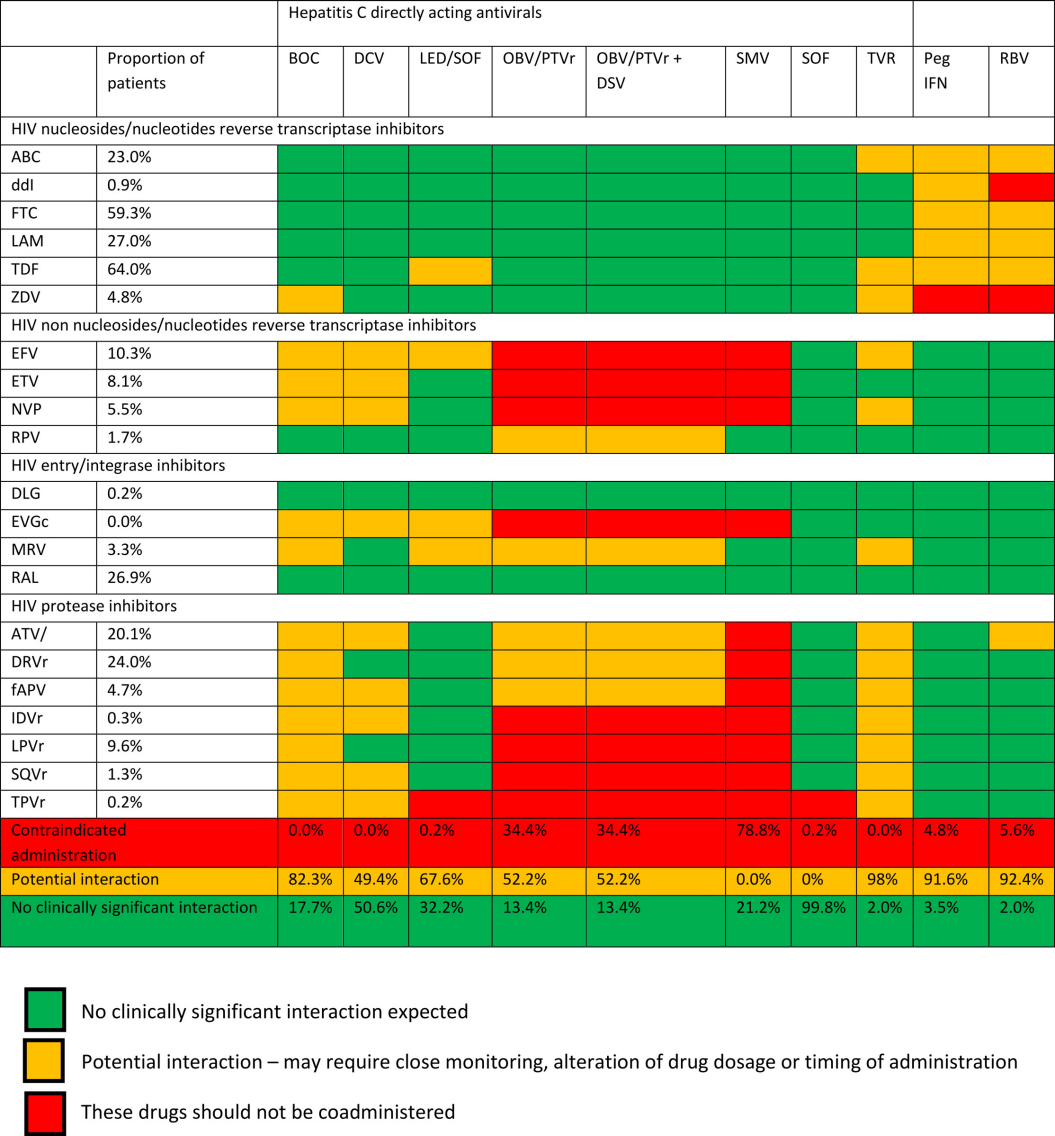
Distribution of antiretroviral drugs received in 1161 HIV/HCV coinfected patients under cART and potential drug-drug interactions between antiretrovirals and currently available DAAs (patients not under antiretroviral treatment, patients under HCV treatment at the time of analysis and patients with HCV reinfection excluded). Adapted from www.hep-druginteractions.org (Abbreviations: ABC abacavir; ddI didanosine; FTC emtricitabine; LAM lamivudine; TDF tenofovir; ZDV zidovudine; EFV efavirenz; ETV etravirine; NVP nevirapine; RPV rilpivirine; DLG dolutegravir; EVGc cobicistat boosted elvitegravir; MRV maraviroc; RAL raltegravir; ATV atazanavir; ATVr ritonavir boosted atazanavir; DRVr ritonavir boosted darunavir; fAPVr ritonavir boosted fosamprenavir; IDVr ritonavir boosted indinavir; LPVr ritonavir boosted lopinavir; SQVr ritonavir boosted saquinavir; TPVr ritonavir boosted tipranavir; BOC boceprevir; DCV daclatasvir; LED Ledipasvir; OBV ombitasvir; PTVr ritonavir boosted paritaprevir; DSV dasabuvir; SMV simeprevir; SOF sofosbuvir; TVR telaprevir; PegIFN Peg interferon alpha; RBV ribavirin.)

Fig 2 presents the distribution of antiretroviral drugs in patients with a detectable plasma HCV-RNA at the time of analysis and potential drug-drug interactions between these drugs and the currently available DAAs. Combinations of tenofovir or abacavir and emtricitabine or lamivudine were prescribed in almost all patients, while 0.9% and 4.8% were still receiving older NRTIs such as didanosine and zidovudine. Most patients were receiving bPI, mainly boosted darunavir (24%) and boosted atazanavir (20.1%), but 16.1% were receiving other bPI. Efavirenz and etravirine were prescribed in respectively 10.3% and 8.1% of patients while rilpivirine which became available in France at the end of 2012 only, was prescribed in 1.7% of patients at that time. INSTI were prescribed in 27.1% of patients, mostly raltegravir since cobicistat boosted elvitegravir and dolutegravir became available in France in 2013 and 2015, respectively.

When considering potential interactions between the antiretroviral drugs received by the patients and DAAs available in 2015 in France, almost all patients could have received sofosbuvir without modification of the antiretroviral treatment, alteration of drug dosage, or close monitoring, 50.6% could have received the ledipasvir/sofosbuvir fixed-dose combination and 32.2% could have received daclatasvir under the same conditions. However, an additional 49.4% and 67.6% of patients could have received ledipasvir/sofosbuvir or daclatasvir, respectively with adequate adaptation of antiretroviral drugs or close monitoring of the renal function when ledipasvir/sofosbuvir was to be associated with tenofovir. On the other hand, coadministration of the current cART could not have been managed without modification of at least one antiretroviral drug in association with ombitasvir/ritonavir boosted paritaprevir, ombitasvir/ritonavir boosted paritaprevir/dasabuvir and simeprevir in respectively 34.4%, 34.4% and 78.8% of the patients. No clear differences were observed between naïve and pre-treated patients regarding potential drug-drug interactions.

### Potential for drug substitutions within the same class

Most of the contraindications observed with simeprevir were related to the presence of a bPI in the antiretroviral treatment, and less frequently to the presence of a contraindicated NNRTI. When combined with NRTIs only, efavirenz and nevirapine could probably be safely switched to rilpivirine. Such substitutions would only reduce the proportion of patients with contraindicated associations with simeprevir from 78.8% to 65.2%.

Similarly, contraindications with the ombitasvir/ritonavir boosted paritaprevir regimens were either related to the presence of a contraindicated NNRTI or to a contraindicated bPI. When combined with NRTIs only, boosted indinavir, saquinavir or lopinavir could probably be switched to boosted atazanavir or darunavir. Such NNRTI or bPI substitutions would reduce the proportion of patients with contraindicated associations with ombitasvir/ritonavir boosted paritaprevir regimens from 34.4% to 12.2%.

## Discussion

This study describes the profile and management of HIV/HCV coinfection in a large multicenter cohort of patients in 2012. The Dat’AIDS cohort weighted approximately 11% of the estimated 149,900 HIV-infected patients living in France in 2010, and 15% of the 111,500 patients under care [23]. The 15.1% HCV prevalence observed in this study appears similar to the prevalence observed in other data sets [2], indicating that this cohort may be considered as representative of the HIV/HCV coinfected population in France. The main findings were (i) the effective immune and viral control of HIV infection in HIV/HCV coinfected patients, (ii) the high level of previous HCV treatment before 2012, (iii) the predominance of HCV-genotype-1, particularly in treatment-experienced patients, (iv) the presence of a severe fibrosis or cirrhosis in nearly half of the patients and (v) the high level of potential drug-drug interactions between antiretroviral treatment and most DAAs in a large proportion of patients.

With a rate of 64.4% of patients previously treated for HCV or receiving treatment at the time of analysis, this study stresses the high level of access to HCV treatment in France. This result is consistent with data of a recent French survey of profile and care of HIV/HCV coinfected patients, reporting 62% of patients being previously treated or receiving treatment in 2013[2]. Significantly lower rates of treatment uptake have been reported in Canada (25%) [24], the United States (13–23%) [25–27], and Europe (25%-30%) [28, 29]. Differences between health care systems, limited access to hepatologists, as well as tolerability issues of previous HCV therapies may explain such a geographical difference. Given the high expectancies for better tolerated and more efficacious IFN-free regimens now available, an increasing rate of treatment uptake and HCV eradication in this relatively closed HIV/HCV population is expected in the next few years.

Similarly to HCV monoinfected patients in France, HCV genotype 1a largely predominated in our population. However, HCV genotype 4 was the second most frequent genotype, probably reflecting the increasing incidence of acute or recent HCV infection in HIV-infected men having sex with men [30, 31].

A fibrosis score consistent with severe fibrosis (F3) or cirrhosis (F4) was found in 44.9% of the patients with an available fibrosis evaluation and in 54.8% of pre-treated patients. On the other hand, a fibrosis score consistent with the absence of fibrosis (F0) or with mild fibrosis (F1) was observed in 44.5% of patients and in 53.2% of naïve patients. This high percentage of severe fibrosis and cirrhosis in this population emphasizes the pertinence of the European guidelines recommending that HCV treatment should be prioritized regardless of the fibrosis stage.

Almost all patients were receiving cART at the time of the study but only 16.7% received combinations of antiretroviral drugs with no or limited interactions with the currently available DAAs (15.4% combinations of 2 NRTIs with 1 INSTI and 1.3% combinations of 2 NRTIs with rilpivirine) [19]. Sofosbuvir and the fixed-dose sofosbuvir-ledipasvir combination could have been used without dose adjustment in almost all patients. The only requirement for the latter combination being a close monitoring of the renal function in two thirds of the patients, due to the increase in tenofovir AUC expected when combining both drugs [18, 19]. Similarly, daclatasvir could have been prescribed in almost all patients but would have required a dosage adjustment of NNRTI or bPI in 49.4% of the patients. Conversely, associations with the 2D/3D regimens (ombitasvir/ritonavir boosted paritaprevir with or without dasabuvir) would have required either a switch of drugs within the same class (e.g. switching efavirenz for rilpivirine or boosted lopinavir to atazanavir or darunavir), a scheme adjustment of bPI in 52% of the patients or a switch of the drug class (e.g. switching a bPI or a NNRTI to an INSTI) in another 34.4%. Simeprevir appears to be the most difficult DAA to combine with antiretroviral drugs, requiring a switch in the class of drugs in 78.8% of the patients. A similar high rate of 88.4% of potential contraindicated drug-drug interactions has been previously reported when theoretically adding simeprevir to the current treatment in a US HIV/HCV coinfected cohort [32]. Such results appeared in sharp contrast with the 12.6% of potential contraindicated drug-drug interactions demonstrated by the same team when theoretically adding simeprevir to the current treatment in a US HCV monoinfected cohort [33]. Therefore, sofosbuvir/ledipasvir and sofosbuvir/daclatasvir combined or not with ribavirin appeared the most suitable combinations in our population. According to the US and European guidelines, no dosage adjustment is required in patients with mild to moderate renal impairment (CrCl 30–80 mL/min) when using sofosbuvir, simeprevir or ledipasvir/sofosbuvir. However, sofosbuvir should not be administered to patients with eGFR <30 ml/min/1.73m². Our data (Table 1) indicate that only 1.3% of patients have a creatinine clearance below 30 mL/min indicating that sofosbuvir contraindication only affects a minority of our patients.

Substitution of a molecule with risk of interaction within the same class of drug is mainly limited to patients receiving standard treatment combining NRTI with NNRTI or bPI. Such substitutions would limit contraindicated associations with ombitasvir/ritonavir boosted paritaprevir and ombitasvir/ritonavir boosted paritaprevir/dasabuvir but would have a limited impact regarding simeprevir. Conversely, switch from one class of drug to another would require at least an analysis of HIV resistance. This analysis is of particular importance since most of our patients had received multiple cART combinations previously. The difference in cART distribution observed between the 1196 untreated patients and the 132 receiving HCV treatment at the same time may illustrate the feasibility and limit of such a switch. However, one cannot exclude the possibility that the first patients receiving first-generation HCV protease inhibitors in 2012 were selected partly because of their ability to combine the available DAA at that time with cART.

Finally, nearly 25% of the patients were receiving complex associations of antiretroviral drugs, usually combining 3 or more classes of drugs. Assessing whether or not the antiretroviral treatment of these patients could be modified to a treatment with less potent drug-drug interactions would require, through a multidisciplinary approach, a complex analysis of the full virological history of each patient, including toxicity and resistance issues. This proportion of 25% is of the same magnitude as that from Cope et al. who reported that safe switch was not possible in 19% of cases [34].

Our study presents several limitations. History of decompensated cirrhosis or other causes of liver disease identified in HIV-infected patients, such as NASH or drug-induced liver injury, was not collected as well as other comorbidities, including renal failure. Concomitant treatment for comorbidities was not collected, which could have increased potential drug-drug interactions. Values of markers such as plasma albumin, bilirubin or prothrombin time were not available, making Child-Pugh and MELD scores not possible to be determined. The methods used for the evaluation of fibrosis scores were heterogeneous, but all were validated. Finally, the distribution of antiretroviral drugs could have slightly changed since completion of the data, given the availability of new antiretrovirals or combinations of antiretrovirals. However, considering the median duration of HIV infection of 21 years in this population, it appears unlikely that the availability of these new drugs could have substantially modified the pattern we describe here.

In conclusion, HIV infection appeared well controlled in this population of HIV/HCV coinfected patients. Significant potential drug-drug interactions are expected between antiretrovirals and the currently available DAAs in the majority of the patients, as opposed to HCV monoinfected patients. American and European guidelines now recommend that HIV/HCV coinfected patients should be treated the same way as HCV monoinfected patients. Due to the complexity of potential drug-drug interactions and the high percentage of patients in whom such interactions are expected, a close collaboration between hepatologists and HIV/AIDS specialists appears mandatory for an optimal management of these still difficult-to-treat patients.

## Supporting Information

S1 Table Demographics and baseline characteristics in HIV/HCV coinfected and HIV-monoinfected patients in the French DAT’AIDS cohort. (PDF)

S2 Table Last available fibrosis assessment with breakdown according to the mode of assessment (LB, liver biopsy; FS, Fibroscan®; FT, Fibrotest®). (PDF)
