# Supplementary material for: Potential for Drug-Drug Interactions between Antiretrovirals and HCV Direct Acting Antivirals in a Large Cohort of HIV/HCV Coinfected Patients
Source: PLoS One. 2015 Oct 21;10(10):e0141164. doi: 10.1371/journal.pone.0141164 (PMC4619009; doi:10.1371/journal.pone.0141164)
Supplement: S1 Table — (PDF) [file pone.0141164.s001.pdf]

Supporting Table 1: Demographics and baseline characteristics in HIV/HCV coinfected and HIV-monoinfected patients in the French DAT'AIDS cohort.

	HIV monoinfected patients (n= 14,123)	HIV/HCV coinfected patients (n= 2,511)	*p*
Male, n (%)	10,030 (71.0)	1,800 (71.7)	0.498
Age (years), median [IQR]	46.0 [39.0-54.0]	49.0 [45.0-53.0]	<0.001
HIV transmission risk group, n (%)			<0.001
- heterosexual	6,159 (43.6%)	513 (21%)	
- homo/bisexual	6,373 (45.1%)	396 (16%)	
- IVDU	129 (0.9%)	1,345 (54%)	
- other/unknown	1462 (10.3%)	257 (10.2%)	
Alcohol consumption, n (%)	n=10170	n=2157	<0.001
- None	4959 (48.8%)	792 (36.8%)	
- Former	431 (4.2%)	341 (15.8%)	
- Current	4780 (47.0%)	1024 (47.5%)	
Current alcohol intake, n (%)	n=4780	n=1024	<0.001
- <20 g/day	3587 (35.3%)	639 (29.6%)	
- 20-50 g/day	870 (8.6%)	239 (11.1%)	
- >50 g/day	323 (3.2%)	146 (6.8%)	
Smoking, n (%)	n=10949	n=2241	<0.001
- None	4833 (44.1%)	373 (16.6%)	
- Former	2063 (18.8%)	375 (16.7%)	
- Current	4053 (37.0%)	1493 (66.6%)	
Substance abuse, n (%)	n=8013	n=1876	<0.001
- None	7091 (88.5%)	666 (35.5%)	
- Former	384 (4.8%)	667 (35.6%)	
- Opiate substitution	32 (0.4%)	266 (14.2%)	
- Current	506 (6.3%)	277 (14.8%)	
Follow-up of HIV infection (years), median [IQR]	11.0 [5.0-18.0]	21.0 [15.0-25.0]	<0.001
CDC Stage C, n (%)	3,074 (21.8)	692 (27.6%)	<0.001
Under cART, n (%)	13,232 (93.7)	2,424 (96.5%)	<0.001
Nadir CD4 (cells/mm^3), median [IQR]	226 [106-338]	166 [74-259]	<0.001
Current CD4 (cells/mm^3), median [IQR]	587 [420-778]	561 [368-780]	<0.001
Plasma HIV-RNA < 50 copies/mL, n (%)	11,368 (87.7%)	2,092 (88.0%)	0.454
cART exposure (years), median [IQR]	9.0 [3.0-15.0]	15.0 [9.0-17.6]	<0.001
Number of cART lines, median [IQR]	4 [2-7]	6 [3-10]	<0.001
cART naive patients, n (%)	703 (5.0%)	55 (2.2%)	<0.001

CDC Centers for Diseases Control; cART combined antiretroviral treatment; IQR interquartile range, IVDU intravenous drug use.
