# Supplementary material for: Potential for Drug-Drug Interactions between Antiretrovirals and HCV Direct Acting Antivirals in a Large Cohort of HIV/HCV Coinfected Patients
Source: PLoS One. 2015 Oct 21;10(10):e0141164. doi: 10.1371/journal.pone.0141164 (PMC4619009; doi:10.1371/journal.pone.0141164)
Supplement: S2 Table — (PDF) [file pone.0141164.s002.pdf]

Supporting Table 2: Last available fibrosis assessment with breakdown according to the mode of assessment (LB, liver biopsy; FS, Fibroscan®; FT, Fibrotest®)

	Naive patients (n = 525)			Treatment-experienced patients (n = 443)		
n *(%)*	LB	FS	FT	LB	FS	FT
F0-F1	22 *(53.7)*	212 *(60.9)*	73 *(38.2)*	9 *(14.1)*	132 *(44.9)*	18 *(13.8)*
F2	12 *(29.3)*	95 *(27.3)*	49 *(25.7)*	22 *(34.4)*	88 *(29.9)*	39 *(30.0)*
F3	2 *(4.9)*		25 *(13.1)*	16 *(25.0)*		22 *(16.9)*
F4	5 *(12.2)*	41 *(11.8)*	44 *(23.0)*	17 *(26.7)*	74 *(25.2)*	51 *(39.2)*
Total	41	348	191	64	294	130

55 naive patients and 45 treatment-experienced patients had multiple evaluations of fibrosis at the same time.

Cut-off for Fibroscan® values: ≤7 kPa: F0-F1; 7-14.5 kPa: F2-F3; ≥14.5 kPa: F4

Cut-off for Fibrotest® values: ≤0.31: F0-1; 0.32–0.58: F2; 0.59–0.72: F3; 0.73–1.00: F4
